# An Ancient Origin for the Enigmatic Flat-Headed Frogs (Bombinatoridae: *Barbourula*) from the Islands of Southeast Asia

**DOI:** 10.1371/journal.pone.0012090

**Published:** 2010-08-09

**Authors:** David C. Blackburn, David P. Bickford, Arvin C. Diesmos, Djoko T. Iskandar, Rafe M. Brown

**Affiliations:** 1 Biodiversity Institute, and Department of Ecology and Evolutionary Biology, University of Kansas, Lawrence, Kansas, United States of America; 2 National University of Singapore, Singapore, Singapore; 3 National Museum of the Philippines, Ermita, Manila, Philippines; 4 Institut Teknologi Bandung, Bandung, Java, Indonesia; Natural History Museum of Denmark, Denmark

## Abstract

**Background:**

The complex history of Southeast Asian islands has long been of interest to biogeographers. Dispersal and vicariance events in the Pleistocene have received the most attention, though recent studies suggest a potentially more ancient history to components of the terrestrial fauna. Among this fauna is the enigmatic archaeobatrachian frog genus *Barbourula*, which only occurs on the islands of Borneo and Palawan. We utilize this lineage to gain unique insight into the temporal history of lineage diversification in Southeast Asian islands.

**Methodology/Principal Findings:**

Using mitochondrial and nuclear genetic data, multiple fossil calibration points, and likelihood and Bayesian methods, we estimate phylogenetic relationships and divergence times for *Barbourula*. We determine the sensitivity of focal divergence times to specific calibration points by jackknife approach in which each calibration point is excluded from analysis. We find that relevant divergence time estimates are robust to the exclusion of specific calibration points. *Barbourula* is recovered as a monophyletic lineage nested within a monophyletic Costata. *Barbourula* diverged from its sister taxon *Bombina* in the Paleogene and the two species of *Barbourula* diverged in the Late Miocene.

**Conclusions/Significance:**

The divergences within *Barbourula* and between it and *Bombina* are surprisingly old and represent the oldest estimates for a cladogenetic event resulting in living taxa endemic to Southeast Asian islands. Moreover, these divergence time estimates are consistent with a new biogeographic scenario: the Palawan Ark Hypothesis. We suggest that components of Palawan's terrestrial fauna might have “rafted” on emergent portions of the North Palawan Block during its migration from the Asian mainland to its present-day position near Borneo. Further, dispersal from Palawan to Borneo (rather than Borneo to Palawan) may explain the current day disjunct distribution of this ancient lineage.

## Introduction

In the species-rich islands of Southeast Asia, the temporal patterns of biological diversification remain largely unexplored. Much of the biodiversity in this region is presumed to be the product of geological events and/or climatic processes in the Pliocene or Pleistocene [1]–[3]. Yet several recent studies suggest an older history to components of this diverse flora and fauna [4]–[6]. Understanding the temporal patterns of faunal diversification in this geologically complex region might require careful re-evaluation of previous hypotheses of historical biogeography. We present the first molecular phylogenetic analysis for the frog genus *Barbourula* and reveal a surprisingly ancient origin consistent with a largely ignored biogeographic hypothesis for one island in Southeast Asia.

Among living frogs, few have remained as enigmatic as the genus *Barbourula*. Since its description by Taylor & Noble [7], *Barbourula* has been known from few specimens and relatively few localities, and little to nothing is known of its natural history or reproductive mode [8]. Adding to its unusual reputation, *B. kalimantanensis* was recently discovered to be the only frog species lacking lungs [9]. The two known species of *Barbourula* are endemic to the Southeast Asian islands of Borneo (*B. kalimantanensis*) and Palawan (*B. busuangensis*). Morphological studies suggest that *Barbourula* is closely related to the genus *Bombina* from Europe and eastern Asia [10]–[13]. The distributions of these two genera, with one endemic to the islands of Southeast Asia and the other restricted to the Laurasian mainland, make this lineage particularly interesting for addressing temporal and spatial patterns of diversification in Southeast Asia.

**Figure 1:**
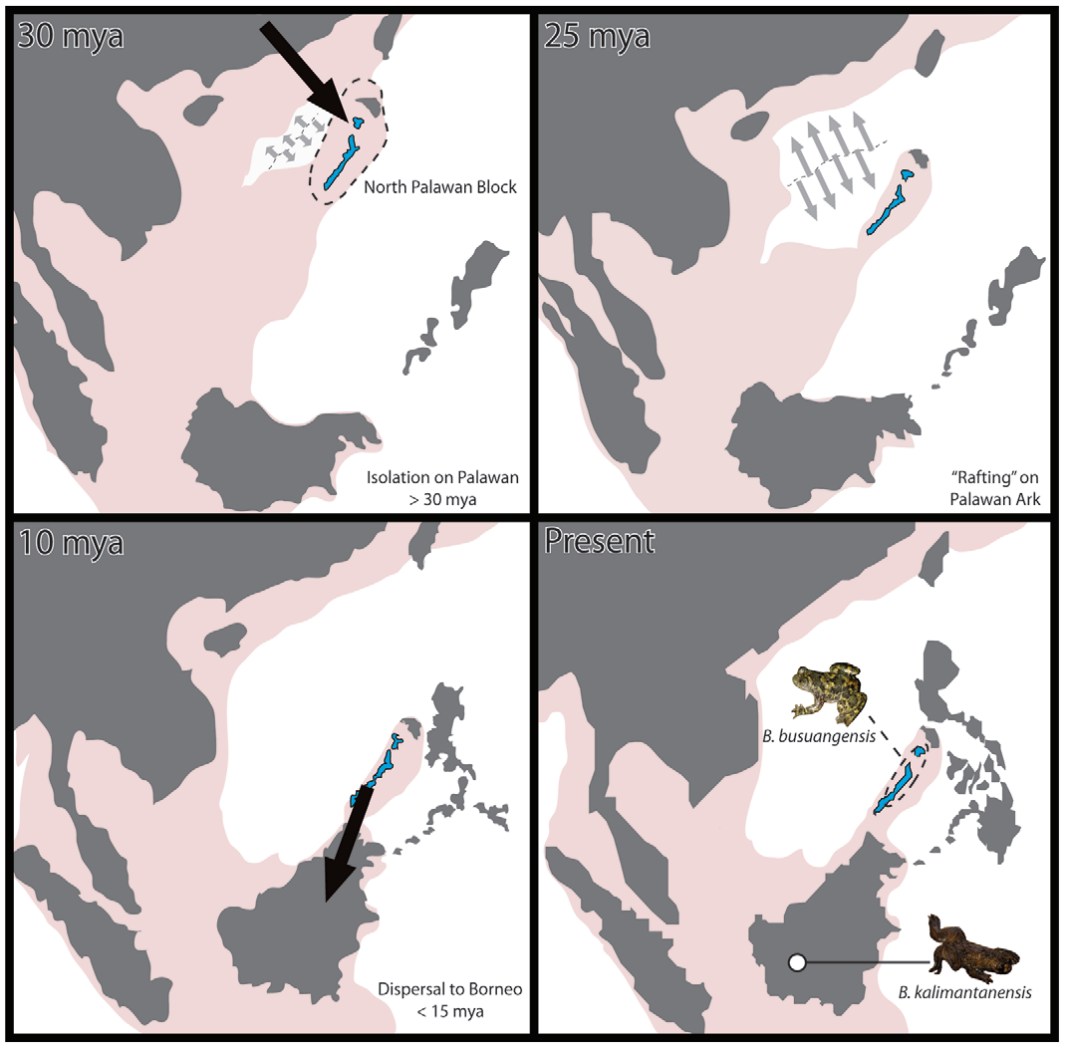
Proposed “Palawan Ark Hypothesis” based on reconstructions of geological history of Southeast Asia [**16**]. Pale red represents submarine continental margins. Large black arrows indicate proposed dispersal routes; small gray arrows indicate sea-floor spreading.

Several hypotheses, couched in a biogeographic framework, exist for the timing of divergence between *Barbourula* and *Bombina*. Savage [14] proposed a pre-Oligocene divergence between these genera on the Asian mainland, but that adaptation to tropical habitats led *Barbourula* to be subsequently restricted to the Sunda Shelf. Inger [15] later suggested that this divergence might have occurred as early as the late Mesozoic. No explicit hypotheses exist for the timing of divergence within *Barbourula*, though this presumably occurred when Palawan was in proximity to Borneo [6], [16]. We offer a new biogeographic scenario, the ‘Palawan Ark Hypothesis’ (Fig. 1), that combines aspects of the above: (1) divergence between *Barbourula* and *Bombina* is correlated with and/or precedes isolation of *Barbourula* on Palawan with the opening of the South China Sea (>30 mya; [6], [16]); and (2) divergence between *B. busuangensis* and *B. kalimantanensis* occurred when Palawan was near to Borneo (<15 mya; [16]). Significantly, support for this hypothesis would suggest that some portion of Palawan has been above water since the Late Paleogene. In this study, we estimate the phylogenetic relationships of *Barbourula* based on novel data from the mitochondrial and nuclear genomes. Then, with estimates of divergence times between *Barbourula* and *Bombina* and within *Barbourula*, we evaluate these biogeographic hypotheses.

## Materials and Methods

### Ethics Statement

Permissions to collect and export specimens were obtained by the necessary agencies in Borneo and the Philippines: Philippine Department of Environment and Natural Resources, Palawan Council for Sustainable Development, Indonesian State Ministry of Research and Technology (RISTEK), and Bukit Baka-Bukit Raya National. Protocols for components of this research were approved by the University of Kansas IACUC (# 158-02).

### Data Collection

To determine the phylogenetic relationships, DNA sequence data were collected from three specimens of *Barbourula*: two of *Barbourula busuangensis* (KU 308965, 324598) and one of *B. kalimantanensis* (RMBR 1117). Genomic DNA was extracted from tissue samples using a guanidine thiocyanate method following the protocol of Esselstyn *et al.* [17]. Polymerase chain reaction (PCR) was used to amplify a region of mitochondrial DNA (∼2000 bp) comprising genes encoding for 12S and 16S ribosomal RNA and the intervening valine transfer RNA. Three nuclear genes (CXC chemokine receptor 4–*CXCR4*; sodium-calcium exchanger 1–*NCX1*; solute carrier family 8 [sodium/calcium exchanger], member 3–*SLC8A3*) were also amplified by PCR; new primers were designed for two nuclear genes (*NCX1*, *SLC8A3*) based on previously published data (e.g., [18], [19]). All primer details are provided in Table 1. The PCR conditions used were standard and the thermal cycle profile was as follows: 94°C (3 min; 35 cycles of 94°C (30 s), 50°C [for mt genes] or 55°C [for nuc genes] (30 s), 72°C (1 min); 72°C (7 min). Purification and sequencing follows Esselstyn *et al.* [17]. Consensus sequences were generated in Sequencer v.4.7 (Gene Codes Corporation) and manually vetted. Resulting sequence lengths and GenBank accession numbers are provided in Tables 2 and 3.

**Table 1:** Primers used for PCR in this study.

Primer Name	Genome	Directionality	Sequence	Reference
12L1	mt	5′ →	AAAAAGCTTCAAACTGGGATTAGATACCCCACTAT	[96]
16SH	mt	3′ ←	GCTAGACCATKATGCAAAAGGTA	[97]
12SM	mt	5′ →	GGCAAGTCGTAACATGGTAAG	[98]
16SA	mt	3′ ←	ATGTTTTTGGTAAACAGGCG	[99]
16SC	mt	5′ →	GTRGGCCTAAAAGCAGCCAC	[98]
16SD	mt	3′ ←	CTCCGGTCTGAACTCAGATCACGTAG	[98]
CXCR4-G	nuc	5′ →	AGCAACAGTGGAARAANGC	[100]
CXCR4-N	nuc	3′ ←	GGTCATGGGTTATCARAARAARTC	[100]
NCX1-Barb1f	nuc	5′ →	CCCTTATGGCTCTTGGYTC	*This study*
NCX1-Barb1r	nuc	3′ ←	AKCCCARRCWTGCAAGAGGT	*This study*
SLC8A3-Barb1f	nuc	5′ →	ACRTCACAGGARCGAGAGAT	*This study*
SLC8A3-Barb1r	nuc	3′ ←	TCCTTTTGGGTTTCYCCWGA	*This study*

**Table 2:** Lengths of DNA sequences generated in this study (GenBank numbers reported in Table 3).

Taxon	Catalog #	12S–16S (mt)	*CXCR4* (nuc)	*NCX1* (nuc)	*SLC8A3* (nuc)
*B. busuangensis*	KU 308965	2018 bp	723 bp	960 bp	991 bp
*B. busuangensis*	KU 324598	2005 bp	722 bp	1036 bp	998 bp
*B. kalimantanensis*	RMBR 1117	1971 bp	724 bp	1017 bp	980 bp

**Table 3:** GenBank accession and relevant catalog numbers for taxa included in this study.

Taxon	Order	Family	Catalog #	12S–16S (mt)	*CXCR4* (nuc)	*NCX1* (nuc)	*SLC8A3* (nuc)
*Alytes obstetricans*	Anura	Alytidae		DQ283112	AY364170	AY523703	EF107345
*Ascaphus montanus*	Anura	Leiopelmatidae		AY236830	AY523698	AY523730	EF107399
*Ascaphus truei*	Anura	Leiopelmatidae		AJ871087	AY523695	AY523731	AY948893
*Barbourula busuangensis*	Anura	Bombinatoridae	KU 308965	HM769265	HM769268	HM769271	HM769274
*Barbourula busuangensis*	Anura	Bombinatoridae	KU 324598	HM769264	HM769267	HM769270	HM769273
*Barbourula kalimantanensis*	Anura	Bombinatoridae	RMBR 1117	HM769263	HM769266	HM769269	HM769272
*Bombina orientalis*	Anura	Bombinatoridae		EU531351	AY364177	AY523715	AY948867
*Bombina variegata*	Anura	Bombinatoridae		EU531355	AY523693	AY523705	EF107347
*Brachytarsophrys feae*	Anura	Megophryidae		AY236799	AY523690	AY523725	EF107359
*Discoglossus pictus*	Anura	Alytidae		DQ283435	AY364172	AY523708	AY948858
*Heleophryne purcelli*	Anura	Heleophrynidae		AY843593	AY364191	AY948833	AY948892
*Hymenochirus boettgeri*	Anura	Pipidae		AY341700	AY523685	AY523702	EF107344
*Leiopelma archeyi*	Anura	Leiopelmatidae		DQ283215	AY523700	AY523723	EF107408
*Leiopelma hochstetteri*	Anura	Leiopelmatidae		DQ283217	AY523696	AY523734	AY948902
*Leptobrachium montanum*	Anura	Megophryidae		EU180881	AY523688	AY523713	EF107352
*Leptolalax arayai*	Anura	Megophryidae		DQ642119	AY523689	AY523714	EF107353
*Pelobates cultripes*	Anura	Pelobatidae		AY236801	AY364171	AY523707	AY948857
*Pelodytes punctatus*	Anura	Pelodytidae		DQ283111	AY364173	AY523709	AY948859
*Pipa pipa*	Anura	Pipidae		AY581621	AY364174	AY523711	EF107351
*Pyxicephalus edulis*	Anura	Pyxicephalidae		DQ283157	EF107494	EF107274	EF107438
*Rhinophrynus dorsalis*	Anura	Rhinophrynidae		AY581620	AY523699	AY523722	AY948894
*Scaphiopus hurterii*	Anura	Scaphiopodidae		AY236828	AY523692	AY523720	EF107392
*Spea multiplicata*	Anura	Scaphiopodidae		AY236823	AY523701	AY523724	AY948903
*Xenopus tropicalis*	Anura	Pipidae		NC006839	AY523697	AY523721	AY948891
*Proteus anguinus*	Caudata	Proteidae		GQ368659	EF107467	EF107243	EF107402
*Salamandra salamandra*	Caudata	Salamandridae		DQ283440	EF017999	EF018024	EF107368

### Taxon Sampling

In choosing taxa for the phylogenetic analyses, we sought to include genera from all major lineages of extant non-neobatrachian frogs (i.e., Archaeobatrachia *sensu* [20]) as well as representatives of the Neobatrachia. Data are available for the mitochondrial and nuclear genes sequenced for *Barbourula* for every family of archaeobatrachian frog as well as all currently recognized genera except *Pseudhymenochirus* (Pipidae) and a number of megophyrid taxa (*Borneophrys*, *Leptobrachella*, *Megophrys*, *Ophryophryne*, *Oreolalax*, *Scutiger*, and *Xenophrys*). Two representative neobatrachian genera (*Heleophryne*, *Pyxicephalus*) were chosen to span the deepest divergence within Neobatrachia [19], [21], [22]. We are confident that the design of this analysis allows for a rigorous test of the phylogenetic relationships of *Barbourula*. Last, two representative salamander genera were included as outgroups (*Proteus* and *Salamandra*).

### Phylogenetic Analyses

For phylogenetic analysis, newly collected sequences were included in a data matrix with previously published sequences available in GenBank (Table 2). For each gene region, a multiple alignment, comprising DNA sequences of unequal length for 26 terminal taxa, was generated using default parameters in Clustal X v.1.83.1 [23]. The resulting alignment of the mitogenomic region was trimmed such that the 5′ and 3′ positions correspond, respectively, to positions 2478–4540 of the *Xenopus laevis* mitochondrial genome (GenBank NC-001573). Alignment lengths for the nuclear genes used in the analysis were as follows: *CXCR4*–655 bp; *NCX1*–1015 bp; and *SLC8A3*–980 bp.

The combined dataset of 4733 bp was partitioned and the best-fit model of sequence evolution was applied to each partition. The data were divided into ten partitions (Table 4): one partition containing all mitochondrial data, and one for each codon position for each of three nuclear genes. The best-fit model of sequence evolution for each partition was selected using the Akaike information criterion (AIC) in MrModeltest v.2.3 [24]; selected models are presented in Table 4.

**Table 4:** Partitions and models used in phylogenetic analyses.

Gene	Model
12S–16S	GTR + I + Γ
*CXCR4* 1^st^ pos.	GTR + Γ*
*CXCR4* 2^nd^ pos.	GTR + I + Γ
*CXCR4* 3^rd^ pos.	GTR + I + Γ
*NCX1* 1^st^ pos.	GTR + I + Γ
*NCX1* 2^nd^ pos.	GTR + I + Γ
*NCX1* 3^rd^ pos.	GTR + I + Γ
*SLC8A3* 1^st^ pos.	GTR + I + Γ
*SCL8A3* 2^nd^ pos.	HKY + I + Γ
*SLC8A3* 3^rd^ pos.	GTR + Γ

*For the partition corresponding to the first codon position of *CXCR4*, the best-fit model was the SYM substitution model rather than GTR. However, because SYM is a subset of the GTR model in which base frequencies are equal, we decided to estimate the base frequencies from the data. Inspection of the base frequencies estimated during Bayesian analysis revealed that these frequencies are very similar for this partition (data not shown).

We employed both Bayesian and maximum-likelihood (ML) estimates of phylogeny. A Bayesian estimate of phylogeny was obtained using MrBayes v.3.1.2 and the models of sequence evolution selected above for each partition. The Γ rate parameters, proportions of invariant sites, substitution rate matrices, and nucleotide frequencies were unlinked between partitions. Four runs of four MCMC chains were run for 20 million generations, sampled every 2000 generations, using a temperature of 0.2 and default priors. The first two million generations were discarded as burn-in following examination of trends and distributions of log-likelihoods and parameter values using Tracer v.1.4 [25]; effective sample sizes (ESS) from each run were all above 1,500. Convergence was assessed by examining correlations of split frequencies among runs in AWTY [26]. The phylogeny and posterior probabilities were estimated from the post-burn-in trees. Split support was calculated using SumTrees [27] and the maximum clade credibility tree (MCCT—the post-burn-in tree with the maximum product of the posterior clade probabilities) was estimated using TreeAnnotator v.1.5.3 [28]. Topologies with posterior probabilities (PP)≥95% were considered well-supported following [29]. ML analyses were conducted on the aligned sequence data in RAxML v.7.0.4 [30] using a random starting tree, the faster rapid hill-climbing algorithm of [31], and a GTR+Γ+I model of sequence evolution for each partition; similar analyses were conducted with a GTR+Γ model resulting in an identical preferred topology and similar support values (GTR+Γ −ln L = 42591.173; GTR+Γ+I ln L = −42487.327; additional data not shown). One hundred search repetitions of this ML analysis were carried out and the phylogenetic estimate with the smallest −ln likelihood score was used as the preferred ML phylogeny. One thousand nonparametric bootstrap replicates were performed in RAxML using the same partitions and models of sequence evolution with one search replicate per bootstrap replicate and a random starting tree; branch lengths and model parameters were optimized during the bootstrap analysis. Split support was calculated using SumTrees. Nodes present in ≥70% of the bootstrap replicate phylogenies (BS) were considered well-supported following [32].

### Divergence Time Estimation

Divergence times were estimated by generating chronograms using the preferred topology from ML and Bayesian analyses and five fossil calibration points. In contrast to a number of recent studies [18], [19], [22], [33]–[35], we do not use paleobiogeographic calibration points or fossils that have not been included in a cladistic analysis. Amphibians are often presumed to have limited dispersal ability over oceanic barriers. As such, a number of studies of amphibian diversification utilize paleobiogeographic calibrations based on the timing of separation of landmasses as estimates of the minimum divergence time between two taxa or clades. Yet, because so many recent studies have inferred the dispersal of amphibians over oceanic barriers [36]–[44], we believe that such calibration points should be abandoned. We restricted our calibration points to fossil taxa for which taxonomic affinities have been demonstrated by phylogenetic analyses. Thus, a fossil was used to date a particular clade of extant taxa if cladistic analysis suggested that the fossil taxon is part of that clade. Data on fossil calibration points are provided in Table 5. As precise dates (in millions of years) are not available for these calibration points, the dates used are the most recent dates associated with the geological time intervals from which the fossils were collected. In addition, to allow the possibility that stratigraphic assignment of fossil taxa might be incorrect, we relaxed the strict minimum divergence time calibration by using a normal distribution around the minimum date for a particular stratigraphic interval. If biased, it should only underestimate the divergence time; in other words, a poorly dated fossil from the Lower Cretaceous might be 130 million years old, but we would recognize it as being “at least 99 million years old.” We view this methodology as inherently conservative.

**Table 5:** Calibration points used for divergence time analyses.

Calibration	Clade	Fossil Taxon	Est. Age	Calibration Age	Age Reference	Cladistic Analysis
1	Batrachia	*Triadobatrachus*	Early T	245.0 mya	[101]	[102]
2	Costata	*Eodiscoglossus*	Middle J	164.0 mya	[103]	[13]
3	Xenoanura	*Rhadinosteus*	Late J	151.0 mya*	[104]	[104]
4	Neobatrachia	*Arariphrynus*, *Eurycephalella*	Early K	99.0 mya	[105]	[105]
5	Pelobatoidea	*Eopelobates*	Eocene	48.6 mya**	[106]–[107]	[108]

*This date differs from Wiens [22] use of 144 mya because he attributes the fossil to Tithonian rather than Kimmeridgian.

**Whereas Roček and Rage [109] retained the identification by Antunes and Russell [106] of anurans from the lower Eocene of Silveirinha, Portugal as *Eopelobates*, Rage and Augé [107] have cast some doubt over this identification in stating that “identification of *Eopelobates* should rest on cranial bones” (p. 105). As our calibration age for Pelobatoidea, we have used 48.6 mya corresponding to the end of the Ypresian in the Early Eocene. However, if the slightly younger age for a Middle Eocene *Eopelobates* locality is used, or even if the younger age for *Elkobatrachus* [108] was used, we expect that our results would be comparable; this is supported by analyses in which this calibration point is removed from divergence time analyses.

Divergence times were estimated using the uncorrelated relaxed clock method [45] as implemented in BEAST v.1.5.2 [46]. Because the phylogenetic analyses resulted in a single topology (i.e., ML and MCCT tree) with generally high support, we constrained the BEAST analyses to estimate divergence times on only this preferred topology. The Yule speciation prior was used for branching patterns and the partitions and corresponding models of sequence evolution implemented for each partition were the same as the analyses in MrBayes (Table 4). MCMC analyses were run for 50 million generations with MCMC steps and divergence times recorded every 1000 generations. Each fossil calibration point provided a “relaxed” minimum divergence time estimate (see above) and assigned a prior with a normal distribution around a mean that was set to a time corresponding to the most recent age of the stratigraphic level estimated for the fossil based on the relevant literature. We utilized the normal distribution on the calibration priors to allow for potential uncertainty in both cladistic relationships and stratigraphic assignments [47]. We also explored the effect of three different standard deviations for the normal distributions assigned to these calibration priors to (i.e., 1.0, 3.0, 5.0). Convergence and effective sample sizes were assessed in Tracer. The 95% highest posterior density interval (HPD) for divergence times were calculated using TreeAnnotator. To evaluate the effect of priors in the analysis, we also conducted an analysis with all five calibration points (normal distributions with standard deviation of 5.0) but in which there were no sequence data. This resulted in substantially different inferred divergence times and 95% HPD intervals (data not shown), which we take as evidence that the data, rather than the priors, are driving the results of these analyses.

We evaluated whether the estimation of divergence times at three specific nodes were sensitive to the calibration points used by utilizing a jackknife approach. These nodes are: (1) the most recent common ancestor (MRCA) of *Barbourula*, *Bombina*, *Alytes*, and *Discoglossu*s; (2) MRCA of *Barbourula* and *Bombina*; and (3) MRCA of *Barbourula busuangensis* and *B. kalimantanensis*. We conducted analyses in BEAST in which each of the calibration points was removed from the analysis (i.e., five analyses each with four calibration points). Specific combinations are listed in Table 6.

**Table 6:** Divergence time estimates (median and 95% HPD, in mya) for *Barbourula*–*Bombina* and *Barbourula busuangensis*–*B. kalimantanensis*.

Calibrations	St. Dev.	*Barbourula*–*Bombina*	*B. busuangensis*–*B. kalimantanensis*
1, 2, 3, 4, 5	1.0	47.08 (34.67–62.01)	11.57 (6.24–16.88)
1, 2, 3, 4, 5	3.0	47.61 (34.68–61.78)	11.58 (6.24–17.01)
1, 2, 3, 4, 5	5.0	47.12 (34.52–60.56)	10.82 (5.97–16.16)
1, 2, 3, 4	5.0	49.21 (37.40–63.23)	10.78 (6.63–15.61)
1, 2, 3, 5	5.0	47.60 (35.12–63.26)	10.92 (6.21–16.40)
1, 2, 4, 5	5.0	46.48 (34.56–59.57)	10.70 (6.30–15.70)
1, 3, 4, 5	5.0	43.57 (32.51–56.19)	9.54 (5.50–15.12)
2, 3, 4, 5	5.0	48.89 (35.76–63.31)	11.10 (6.03–16.48)

For each analysis listed, the combination of calibration points (numbers corresponding to Table 5) and standard deviations for prior of calibration points are provided.

Results of divergence time analyses were evaluated by examining effective samples sizes and burn-in using Tracer and then calculating the median node heights using TreeAnnotator. The median node heights and 95% HPD were compared for divergences across the tree with specific attention to those between *Barbourula* and *Bombina* and within *Barbourula*.

## Results

### Phylogeny

**Figure 2:**
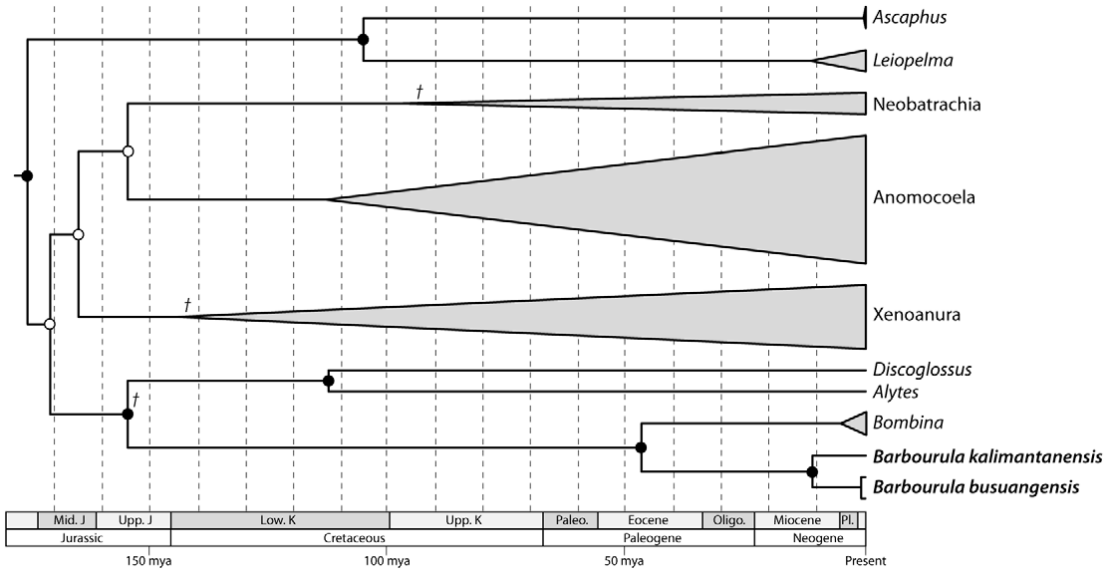
Time-calibrated phylogeny of *Barbourula*. Depicted is the MCCT and ML topology with divergence times (in mya) estimated using all five calibration points and standard deviations of 5.0 for their prior distributions. Nodes are at the inferred median heights with gray bars at selected nodes indicating the 95% HPD. Closed circles indicate high Bayesian and ML support (PP = 1.0; BS>90%); open circles indicate high Bayesian support only. Three of the five divergence time calibration points are indicated by crosses; the remaining two are either within Anomocoela or between Anura and Urodela (outgroup Urodela taxa are not shown; see also Figure S1).

Bayesian and ML analyses resulted in the same preferred phylogenetic topology (MCCT and ML tree) and most nodes receive high support from both Bayesian and ML analyses (Figs. 2, S1). Though the preferred topology from both Bayesian and ML analyses were identical, several nodes related to the placement of the Neobatrachia received low support from ML non-parametric bootstrapping analyses. In addition, the preferred topology from both Bayesian and ML analyses contains a sister-relationship between *Xenopus* and *Hymenochirus*, though this receives low support from both analyses (PP = 0.6, BS = 44%). However, all nodes related to the placement of *Barbourula* received high support, including the Bombinatoridae (*Barbourula*+*Bombina*, PP = 1.0, BS = 100%; *sensu* [48]) and the Alytidae (*Alytes*+*Discoglossus*, PP = 1.0, BS = 100%; *sensu* [21]). Monophyly of the Costata (i.e., Alytidae+Bombinatoridae; *sensu* [21]) is also strongly supported (PP = 1.0, BS = 100%). *Barbourula busuangensis* and *B. kalimantanensis* form a strongly supported clade (PP = 1.0, BS = 100%; Fig. 2) that is sister to the genus *Bombina* (PP = 1.0, BS = 100%).

### Divergence Time Estimation

**Figure 3:**
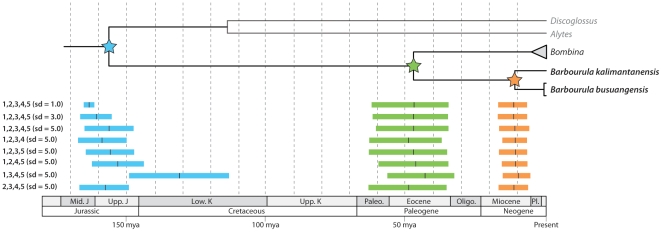
Congruence of divergence times estimated using different calibration points. Graphical representation of the data presented in Table 6; divergence times are given in mya.

Using all five calibration points and the broadest standard deviation for the related priors (5.0), the estimated time of divergence between *Barbourula* and *Bombina* is 47.1 mya (95% HPD: 34.5–60.6 mya). The divergence time between the two species of *Barbourula* is 10.8 mya (95% HPD: 6.0–16.2 mya), whereas the intraspecific divergence within *B. busuangensis* is 0.7 mya (95% HPD: 0.3–1.7 mya). These divergence times are robust to the use of alternative standard deviations for calibration priors and to the exclusion of each calibration point (Table 6; Fig. 3). In summary, median estimates for the divergence of *Barbourula* and *Bombina* range from 43.6–49.2 mya (cumulative 95% HPD: 32.5–63.3 mya) and those between the two species of *Barbourula* range from 9.5–11.6 mya (cumulative 95% HPD: 5.5–17.0 mya).

## Discussion

### Systematics

Our findings that *Barbourula* is monophyletic and that *Barbourula* and *Bombina* are sister taxa (Fig. 2) are not surprising. A close relationship between these genera has been proposed since the discovery of *Barbourula* [7], [10], [12], [15], [49] and has been supported by morphological phylogenetic analyses [11], [13]. In fact, based on morphological comparisons [50], [51], there has been some previous suggestion that *Barbourula kalimantanensis* may be more closely related to *Bombina* than to *B. busuangensis* [51]. However, our analysis provides unequivocal support for the monophyly of *Barbourula*.

Last, the precise relationship of *Barbourula* and *Bombina* to other anurans has long remained uncertain [11], [13], [52]–[59]. Recent molecular phylogenetic studies have unequivocally resolved *Bombina* as the sister to a clade comprising *Alytes* and *Discoglossus* [18], [19], [21], [22], [33], [60], [61]. Our results support this conclusion by inferring *Barbourula* + *Bombina* to be the sister to *Alytes* + *Discoglossus*.

### Divergence Time Estimates

In contrast to the phylogenetic relationships, the estimated divergence times are unexpected as these reveal a great antiquity to the endemism of these Southeast Asian frogs. Divergence between *Barbourula* and *Bombina* occurred in the Paleogene, most likely before the Oligocene (Fig. 2; Table 6). To our knowledge, this is the oldest estimate for a cladogenetic event resulting in living taxa endemic to the islands of Southeast Asia (for comparisons, see [4], [62]–[65]). Similarly, the two species of *Barbourula* diverged in the Late Miocene, thus substantially predating other estimates of divergence between taxa endemic to Borneo and Palawan [5], [6], [66].

The divergence between the two specimens of *Barbourula busuangensis* appears to have occurred during the Pleistocene. These specimens were collected <100 km apart and have an uncorrected *p* distance for the mitochondrial DNA sequence of 1.3%; the nuclear gene sequences for these two specimens are nearly identical. Though admittedly based on little data, this estimated timing of divergence within *B. busuangensis* supports previous assertions that the Pleistocene was an important period for the generation of diversity, including phylogeographic structure, in the Philippines and associated islands [2], [67].

Previous estimates based on molecular data of the divergence time for the last common ancestor of *Barbourula*, *Bombina*, *Alytes*, and *Discoglossus* found a mid-Mesozoic divergence. However, these estimates still differ substantially from one another with some finding this divergence to be in the Lower Cretaceous [22], [68] and others in the Late Triassic or Early Jurassic [19], [33]. Our estimates are broadly consistent with these previous studies by inferring this divergence to be in the mid-Mesozoic (Fig. 2), but still considerably younger (Late Jurassic or Early Cretaceous) than some previous estimates [19], [33]. We believe that these differences are most likely due to the use by other studies of calibration points based on paleobiogeographic events or fossil taxa that have not been demonstrated via cladistic analysis to be nested within the clade to which the calibration was applied. Our results are most consistent with those inferred by Wiens [22] who restricted nine of his ten anuran calibration points to fossils. However, our analysis reveals that such inferences might be led astray even by calibration points based on fossil taxa for which affinities to extant taxa have been demonstrated. For example, in analyses in which the calibration point for the most recent common ancestor of Costata (calibration 2) is removed, divergence time estimates are considerably younger (Fig. 3). This could be due to differing rates of evolution across the phylogeny or error in the inferred phylogenetic relationships of the fossil taxa used. We believe that the latter is more likely in many cases for anurans. In the present case, *Eodiscoglossus* was used to calibrate the origin of the Costata based on the phylogenetic results of Gao & Wang [13], yet it is possible that *Eodiscoglosus* is sister to the clade containing extant members of the Costata. While the removal of fossil calibration points did not affect the divergence times of interest (i.e., *Barbourula*–*Bombina* and within *Barbourula*), this analysis serves to underscore the need to critically evaluate calibration points as well as the importance of divergence estimation methods that incorporate uncertainty, including “relaxed” minimum divergence time calibrations [47].

### Geological Context

Based on evidence supplied by Taylor & Hayes [69] and Holloway [70], Heaney [2] summarized Palawan's geological history in developing a framework for understanding its biogeography. Heaney's [2] summary is as follows:

The islands of the Palawan Arc are a composite of old continental crustal rocks (from the northern half of Palawan Island to southern Mindoro and northern Panay) and more recent fragments of oceanic crust. The continental rocks are Palaeozoic and Mesozoic in age and consist of some clastic sediments and limestone. Limestone sediments containing fossils of marine invertebrates on top of the continental rocks indicate that shallow marine conditions existed from the Jurassic to the Eocene. Recent data suggest a mid-Oligocene to early Miocene (32 to 17 Ma BP) rifting of this material away from the Asian continent [69], [70]; no evidence yet suggests subaerial (emergent) islands before the Miocene. Sedimentary material of Pliocene and Quaternary origin on the edge of the Palawan trench suggests the presence of islands by the Pliocene. The subduction zone has been inactive since the Miocene. (p. 139)

Nearly 25 years later, reconstructions of Palawan's geological history incontrovertibly place at least its northern portion (referred to as the Northern Palawan Block) as originating on the margin of the South China continental crust with rifting between these beginning in the Late Oligocene ([69]–[72]; reviewed in [16]). Recent work suggests that the differences between northern and southern Palawan might be the results of differential uplift and erosion [73] leading to the inference that some portion of southern Palawan may also have a continental origin; indeed, Milsom *et al.* [74] state that continental rocks are widely exposed south of Ulugan Bay. Because of the presence of onshore limestone deposits in northern Palawan ([75] cited in [70]; see also [76]), portions of present-day emergent Palawan are believed to have been below water at some point in the Tertiary (probably as recently as the late Oligocene to early Miocene ([77] cited in [72]). Consequently, it is implied that a terrestrial fauna did not inhabit the island until after this period [2], [78]. Although some portions of northern Palawan were likely below water, there is relatively little evidence for or against the hypothesis that at least some portion of the Palawan continental fragment was above water during the migration of the Northern Palawan Block from the Asian mainland. The complicated biogeography and geology of these islands and high levels of taxonomic distinctiveness of Palawan endemics suggest that northern Palawan may have been an oceanic island before uplift of southern Palawan created an effective bridge to northern Borneo [3], [6], [36], [79]–[84]. Taylor & Hayes [69] suggest that absence of strata from before the mid-Oligocene on the South China margin is consistent with pre-rifting tectonic uplift at this time. Further, reconstructions by Mitchell *et al.* (see Fig. 9 of [72]) suggest that at least some portion of the Northern Palawan Block might have been emergent as early as the Late Eocene as evidenced by clastic deposits. Other information suggesting that emergent portions existed at least by the Early Miocene include evidence of sediments derived from this region during this time period [85] and granite in northern Palawan [86], the origin of which requires a thickened crust that is suggestive of emergent land (R. Hall, pers. comm). Taken together, various geological studies imply both a complicated composition and potentially significant topography in this region preceding the rifting leading to the creation of the South China Sea and the movement of the Northern Palawan Block away from the mainland. Thus, there is the possibility that a portion of this Northern Palawan Block remained continuously above water forming an oceanic island with habitat suitable for terrestrial organisms since possibly the Late Paleogene (see also [87]).

### Biogeography

The estimates of divergence time between *Barbourula* and *Bombina* are consistent with several components of proposed biogeographic hypotheses. Divergence between *Barbourula* and *Bombina* occurred after the Mesozoic but, as proposed by Savage [14], predating the Oligocene, *contra* Inger [88]. Savage [14] hypothesized that *Barbourula* originated in tropical habitats on the Asian mainland and was later restricted to the Sunda Shelf as a consequence of cooling in continental Asia beginning at the Eocene–Oligocene transition [89]–[92]. Yet if *Barbourula* has been a component of the Sunda Shelf fauna for the past 30 million years, then it is anomalous in this fauna by not also occurring on other nearby islands that have been in close association throughout the Cenozoic [16].

As an alternative, we propose that *Barbourula* may have joined the Sunda Shelf fauna following “rafting” on Palawan. This hypothesis is consistent with both the divergence times and the distribution of *Barbourula*. First, the Late Miocene divergence within *Barbourula* is consistent with Palawan moving into close proximity to Borneo at this time [16], [76]. Second, if *Barbourula* colonized Borneo from Palawan, then this lineage has only been part of the Sunda Shelf fauna for the past ∼10 mya rather than the past ∼30+ mya, which might account for its more limited distribution and apparent lack of diversification. Portions of present-day Palawan have been land-positive since at least the Miocene [72] but the evidence for the continuous emergence of one or more component(s) of Palawan since the Eocene is admittedly scarce (though see discussion above). However, our inferred divergence time estimations, especially as these are consistent with both temporal components of the Palawan Ark Hypothesis, suggest that this is at least tenable. As such, we encourage this alternative hypothesis to be further tested with phylogenetic data from other terrestrial organisms, especially those with limited dispersal capabilities.

Notably, our estimate of a Late Miocene divergence between the two species of *Barbourula* fits the geological record well. The Palawan Ark Hypothesis specifically posits dispersal to Borneo if *Barbourula* entered the islands of Southeast Asia via emergent portions of the North Palawan Block. However, this divergence time might be expected regardless of whether the *Barbourula* dispersed from Palawan to Borneo or vice-versa. Curiously, Inger [15] postulated that *Barbourula* might have entered the Philippines during the Miocene or earlier, though the species on Borneo was unknown at that time.

The Palawan Ark Hypothesis posits that *Barbourula* entered the islands of Southeast Asia via emergent portions of Palawan instead of through the Sunda Shelf. Unfortunately, the deep-time geological history of Palawan is too insufficiently known to determine when and for how long portions of the North Palawan Block might have been above water. Given the presence of Eocene limestones on Palawan, it is conceivable that there has never been an emergent landbridge between the Asian mainland and portions of the North Palawan Block. If so, and if *Barbourula* did indeed disperse to the Southeast Asian islands via Palawan, then this would imply an overwater dispersal from the mainland to the emergent portions of the North Palawan Block, possibly as early as the Eocene [72]. Interestingly, such a vicariant event would be congruent with our estimated divergence time between *Bombina* and *Barbourula*, though we admit that this scenario is highly speculative. If the Palawan Ark Hypothesis is not correct, then *Barbourula* is truly a “relict,” having become extinct throughout the Malay Peninsula and Sunda Shelf region through which it must have once dispersed (as posited by [14]). Support for this might be garnered from other taxa occurring on the periphery of the Asian mainland that are sometimes interpreted as relicts of diversification events in warm and/or tropical environments before the Oligocene ([93], [94]; see also [87]).

Most previous studies of Palawan biogeography focus on dispersal between Borneo and the Philippines [2], [67], [81], [95]. Our estimates of divergence times suggest that portions of Palawan might have been above-water since the Paleogene and served as an “ark” carrying fauna and flora from the Asian mainland to its present-day position between the island of Borneo and the Philippines island archipelago.

## Supporting Information

Figure S1 Phylogenetic relationships resolved in this study showing all of the terminal taxa utilized. Depicted is the MCCT and ML topology with divergence times estimated using all six calibration points and standard deviations of 5.0 for their prior distributions (as in Fig. 2). Nodes are at the inferred median heights. Closed circles indicate high Bayesian and ML support (PP = 1.0; BS>100%); for nodes with lower support, BS is provided above the branch and PP below it. Each of the six divergence time calibration points is indicated by a cross.
(5.11 MB TIF)
