# Supplementary figures and images for: An Ancient Origin for the Enigmatic Flat-Headed Frogs (Bombinatoridae: Barbourula) from the Islands of Southeast Asia
Source: PLoS One. 2010 Aug 9;5(8):e12090. doi: 10.1371/journal.pone.0012090 (PMC2918512; doi:10.1371/journal.pone.0012090)

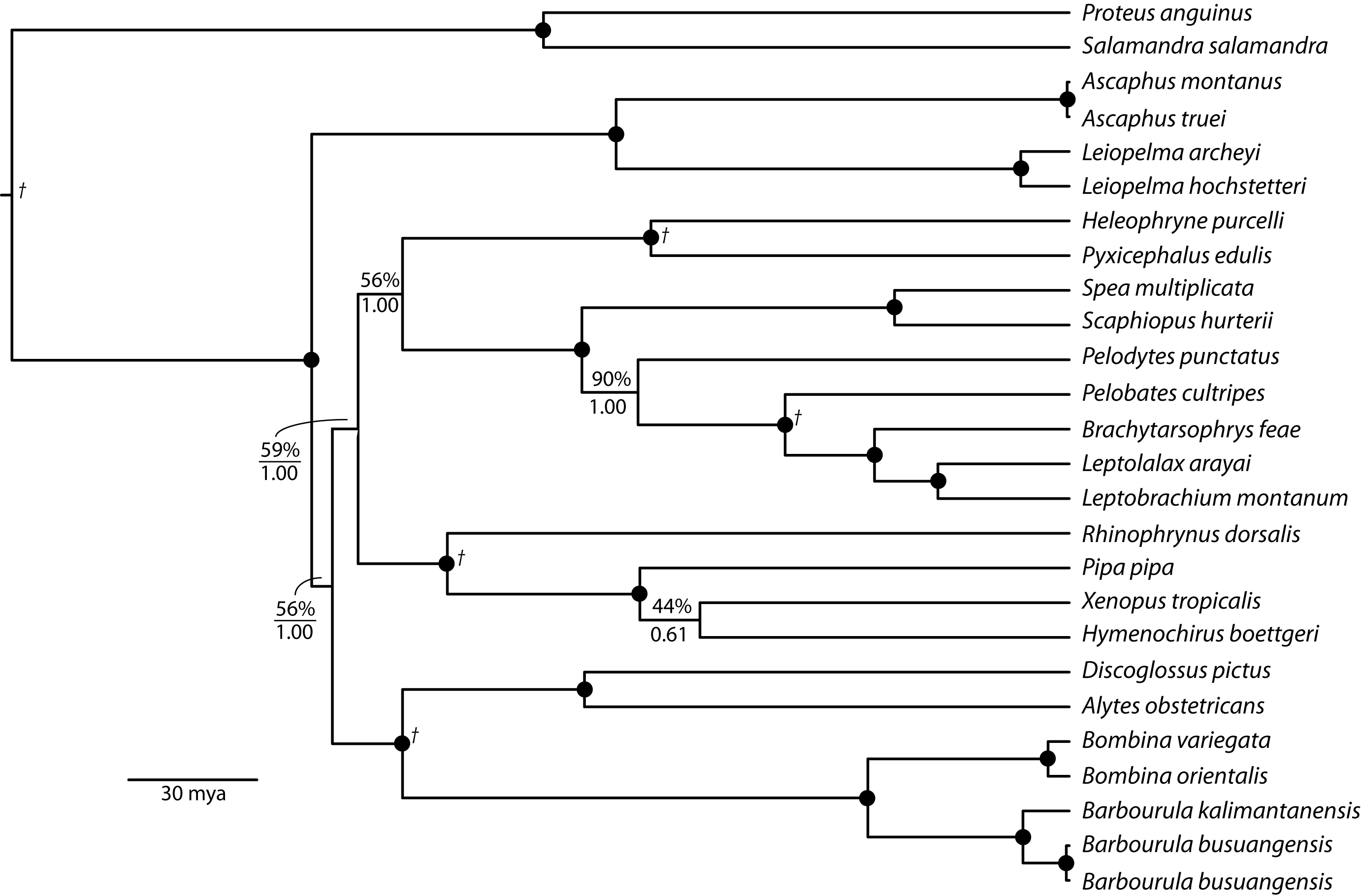

Supplement: Figure S1 — Phylogenetic relationships resolved in this study showing all of the terminal taxa utilized. Depicted is the MCCT and ML topology with divergence times estimated using all six calibration points and standard deviations of 5.0 for their prior distributions (as in Fig. 2). Nodes are at the inferred median heights. Closed circles indicate high Bayesian and ML support (PP = 1.0; BS>100%); for nodes with lower support, BS is provided above the branch and PP below it. Each of the six divergence time calibration points is indicated by a cross. (5.11 MB TIF) [file pone.0012090.s001.tif]
